# Scale-up synthesis of high-quality solid-state-processed CsCuX (X = Cl, Br, I) perovskite nanocrystal materials toward near-ultraviolet flexible electronic properties†

**DOI:** 10.1039/d2ra07100b

**Published:** 2023-02-20

**Authors:** Zhi Jiang, Hezhuang Liu, Jihua Zou, Yixuan Huang, Zhaoquan Xu, Denys Pustovyi, Svetlana Vitusevich

**Affiliations:** a Institute of Biological Information Processing (IBI-3), Forschungszentrum Jülich Leo-Brandtstr 52425 Jülich Germany s.vitusevich@fz-juelich.de z.jiang@fz-juelich.de; b Institute of Fundamental and Frontier Sciences No. 4, Sec. 2, North Jianshe Rd. 610054 Chengdu China

## Abstract

High-quality CsCu_2_X_3_ and Cs_3_Cu_2_X_5_ (X = Cl, Br, I) nanocrystals (NCs) exhibit excellent optoelectronic, physical, and chemical properties for detection of UV radiation due to large carrier mobility and lifetime, and heavy atoms. The nanocrystal materials can be prepared *via* a low-cost and simple solid-state synthesis. However, poor reproducibility and complex synthesis methods of obtaining perovskite NC thin films represent a drawback for the fabrication of the commercial photoelectric device. To address these issues, we develop highly stable CsCu_2_X_3_ and Cs_3_Cu_2_X_5_ NC materials using a facile solid-state reaction method for the scale-up production of halogen lead-free perovskites. We suggest a distinctive way to design a series of nanocrystalline perovskites using short-term synthesis and study the mechanism of perovskite formation using thermal solid-state synthesis. These all-inorganic and lead-free CsCu_2_X_3_ and Cs_3_Cu_2_X_5_ exhibit large photoluminescence quantum yields (PLQYs) up to 95.2%. Moreover, flexible paper photodetectors based on this series of lead-free perovskites show strong photoselectivity and bending stability at 254 nm, 365 nm, and 405 nm wavelengths. High-quality responses with a responsivity of 1.1 × 10^−3^ A W^−1^ and detectivity of 2.71 × 10^9^ jones under UV illumination (10 μW cm^−2^) at a bias voltage of 5 mV are demonstrated. These results open prospects for designing photodetectors, LEDs, and other photosensitive devices.

## Abstract 2

The designed flexible lead-free Cs_3_Cu_2_X_5_ (X = Cl, Br, I) nanocrystal perovskite photodetectors demonstrate excellent properties, including high photoresponses, strong photoselectivity and bending stability at different irradiation wavelengths.

## Graphical abstract

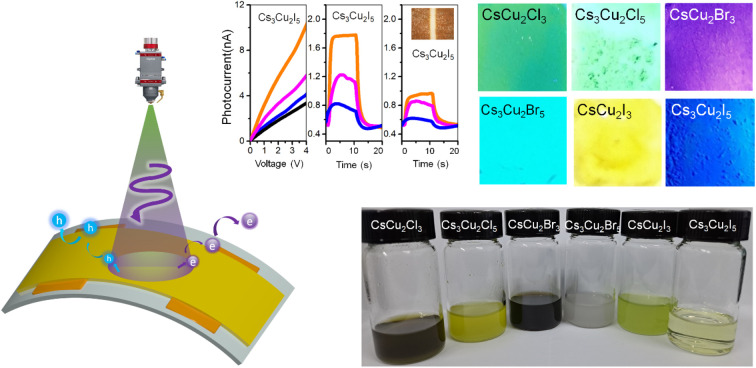

## Introduction

Organic–inorganic metal halide perovskites and formamidinium lead halide (FAPbX_3_) perovskites (PVs) have attracted substantial interest,^1–6^ owing to their excellent optical properties, including superior photoluminescence quantum yields (PLQYs),^5–7^ tunable bandgap,^8^ ease of processability and small exciton binding energy.^9–11^ All-inorganic lead-containing halides CsPbX_3_ (ref. 12) have been widely employed as efficient active materials in solar cells, light-emitting diodes and photodetectors.^13–15^ However, the key issue of these materials is their instability in ambient conditions related to the organic component. On the other hand, the toxicity of lead (Pb) element is another concern because Pb is a hazardous material. As to PV application, electrical characteristics of electronic devices on flexible printable substrate^16^ may deteriorate due to external deformation and surface roughness. The change in device shape may produce surface tension and even defects, which result in weakening the photoelectric properties of photodetectors. Although all-inorganic low-dimensional metal halides demonstrate relatively high thermal stability, photoelectric properties of such materials still suffer from low responsivity (*R*) and detectivity (D*) even at ultra-low operation voltage.^17^

The development of new environmentally-friendly materials without organic lead halide compounds is critical for future photovoltaic and display technologies^18–20^ Numerous groups developed alternative lead-free and organic–inorganic halide perovskites in recent years. Cesium (Cs)-based perovskite nanocrystals (NCs) crystallization technique could utilize suitable solvents to satisfy the need for a diverse variety of crystal geometries and to replace organic materials MA/FA and Pb in the perovskite lattice, such as zero-dimensional (0D) Cs_3_Cu_2_X_5_ NCs^21^ and subsequent crystallization,^22^ inverse temperature crystallization strategy.^23,24^ Unfortunately, lead-free perovskite NCs suffer from another possible obstacle. These organic–inorganic perovskite NCs and photodetectors based on them still suffered from short-term instability under wet conditions, complex process technology, and a small production rate.^25^ It is necessary to develop high-performance, low-cost, gram-scale all-inorganic and lead-free metal halide salts,^26^ using such methods as a hot one-pot solution synthesis^27^ and a solvent-free mechanochemical approach.^28^ A facile all-inorganic halide perovskite solid-state reaction method was reported by Huang^29^ and Roccanova.^30^

The method is useful and promising to synthesize high-quality Cs lead-free copper (Cu) inorganic metal halide PVs. Low-dimensional metal halide perovskites: CsCu_2_X_3_ and Cs_3_Cu_2_X_5_ (X = Cl, Br, I) exhibited fine stability in the atmospheric environment.^31–33^ Although the synthesis and bulk properties of low-dimensional Cs copper halides have been reported, the studies about a wide range of compositional variations of halogen anions and the material application as photodetector on flexible substrates are limited at present.^34,35^ This solid-state reaction technique can be considered as environmentally friendly chemistry because it does not involve the utilization of solvents.

In this paper, orthorhombic single-crystalline lead-free copper halide salts based on CsCu_2_X_3_ and Cs_3_Cu_2_X_5_ are synthesized by reacting stoichiometric amounts of CsX and CuX (X = Cl, Br, I) using solid-state reaction method for the scale-up halogen lead-free perovskites. High-quality properties are demonstrated for PV devices fabricated on a flexible substrate.

## Results and discussion

**Fig. 1:**
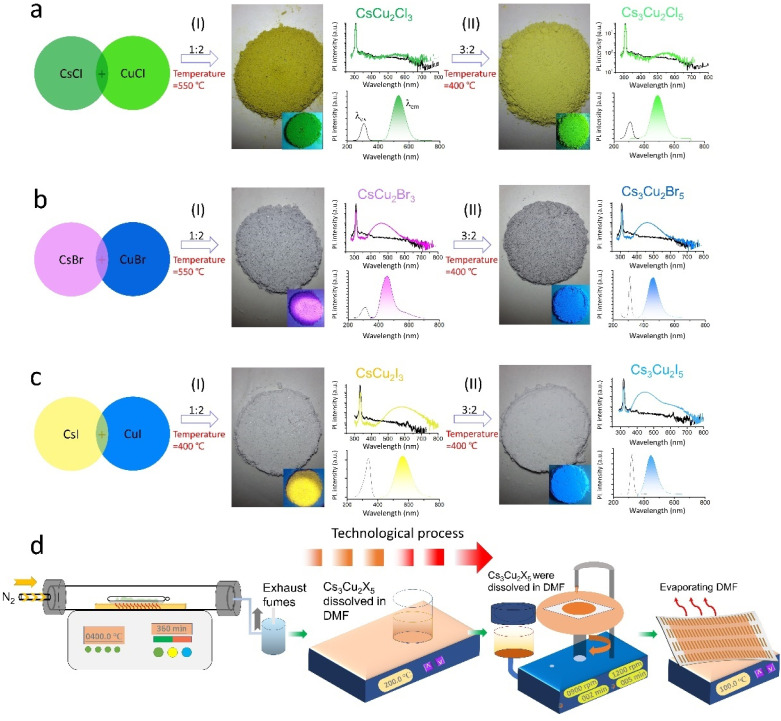
Schematic of typical solid-phase reactions for obtaining all-inorganic lead-free perovskite NC materials, demonstration of excitation of BaSO_4_ line of reference spectrum and emission spectrum, and the corresponding photoluminescence excitation (PLE) and photoluminescence emission (PL). (a) CsCu_2_Cl_3_ and Cs_3_Cu_2_Cl_5_ NCs, (b) CsCu_2_Br_3_ and Cs_3_Cu_2_Br_5_ NCs, (c) CsCu_2_I_3_ and Cs_3_Cu_2_I_5_ NCs. (I) Images of perovskite powder under ambient light (large grey/dark yellow circles) and (II) UV light irradiation (small color circles), the scale bars of 1 cm. (d) Schematic presentation of the fabrication process for thin-film perovskite NC devices with electrode array on the paper.

**Fig. 2:**
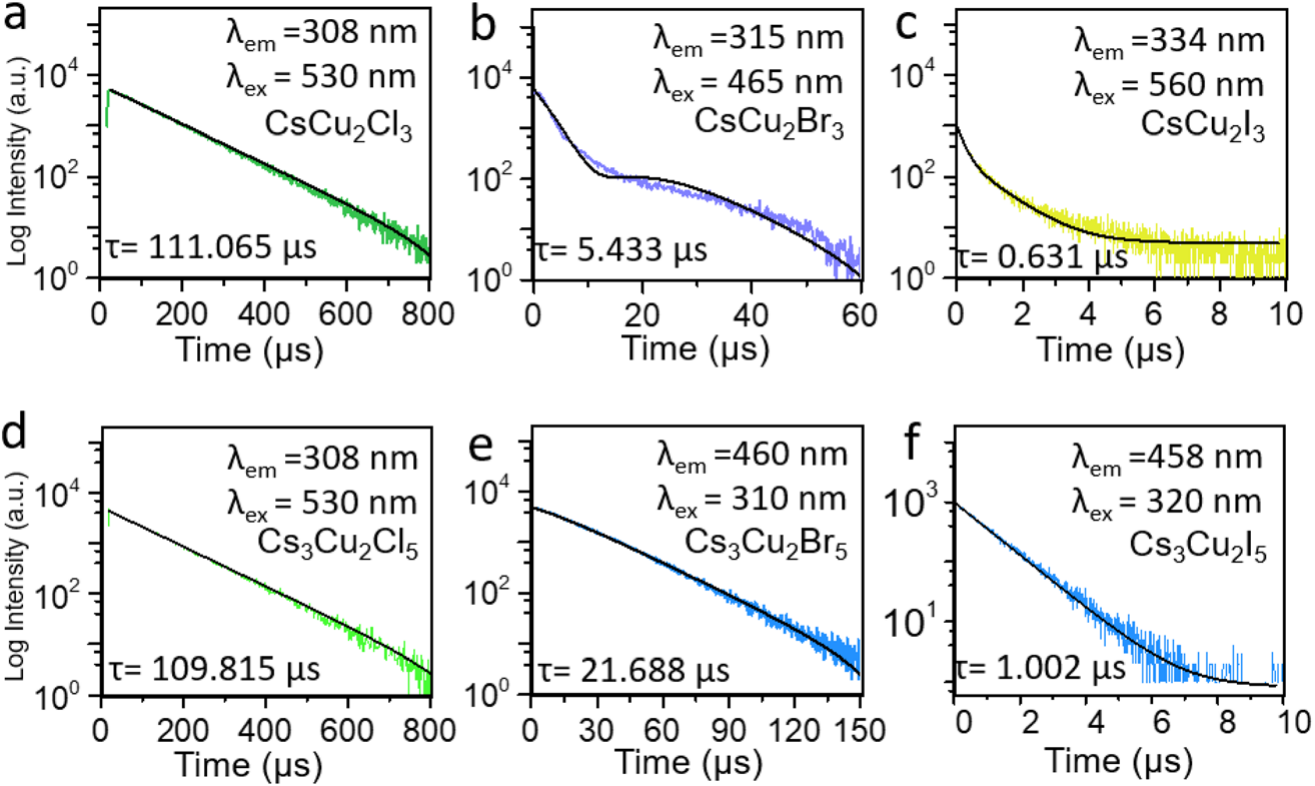
Photoluminescence decays of six lead-free perovskite (a) CsCu_2_Cl_3_ (b) Cs_3_Cu_2_Cl_5_ (c) CsCu_2_Br_3_ (d) Cs_3_Cu_2_Br_5_ (e) CsCu_2_I_3_ (f) Cs_3_Cu_2_I_5_ NC materials. Excitation source = EPL-405 pulsed diode laser, rep rate = 100 kHz, *λ*_em_ = 780 nm, Δλ_em_ = 10 nm.

**Fig. 3:**
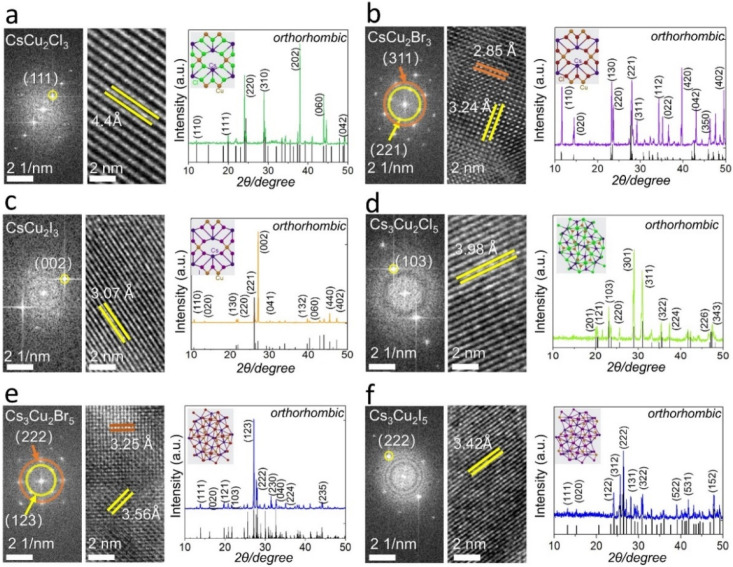
FFT images, HRTEM lattice planes, powder X-ray diffraction pattern of orthorhombic (a) CsCu_2_Cl_3_, (b) CsCu_2_Br_3_, (c) CsCu_2_I_3_, (d) Cs_3_Cu_2_Cl_5_, (e) Cs_3_Cu_2_Br_5_, and (f) Cs_3_Cu_2_I_5_ NC materials. The corresponding crystal systems are shown in the insets.

All-inorganic lead-free perovskites were synthesized *via* a thermal solid-state method, as schematically illustrated in Fig. 1a. The details of growth procedure and synthesis procedure are given in the Experimental section. Briefly, the raw materials were mixed in a glovebox and then after temperature optimization calcined at 400 °C (except CsCu_2_Br_3_ material, which was calcined at 550 °C) for 6 h in a tube furnace to obtain ultra-stable NC materials. This step is finalized by the natural cooling process. The NC films were subsequently fabricated by spin coating the precursor dissolved in *N*,*N*-dimethylformamide (DMF), deposited on a paper substrate and annealing at 100 °C Fig. 1d. By precisely controlling the stoichiometric ratio of CsX : CuX powders, the high-quality single crystalline CsCu_2_X_3_ and Cs_3_Cu_2_X_5_ nanocrystals (NCs) can be obtained, as displayed results in Fig. 1–3. A clear colour change (including green, blue, violet and yellow) over the entire visible range could be controlled owing to the variation of optical absorbance and PL emission. These different photoluminescence spectra emitted from various PV NC powders under UV illumination indicate the formation of tunable bandgaps *via* facile composition engineering.

Starting with the CsCu_2_Cl_3_ and Cs_3_Cu_2_Cl_5_ NC materials in Fig. 1a, it was demonstrated that the color of the PL emission (*λ*_em_) systematically shifts from green (CsCu_2_Cl_3_: 530 nm) to deep green (Cs_3_Cu_2_Cl_5_: 495 nm), from violet (CsCu_2_Br_3_: 435 nm) to blue (Cs_3_Cu_2_Br_5_: 460 nm) and from yellow (CsCu_2_I_3_: 560 nm) to light green (Cs_3_Cu_2_I_5_: 445 nm) in Fig. 1b and c, respectively. NCs can be assigned to an orthorhombic system but different space groups, which is consistent with FFT results.^14^ To gain further insights into the optical properties of as-prepared PV NCs, the time-resolved PL (TRPL) and PLQYs were applied. PLQYs can be related to the defects in the materials, as one can find in Fig. 2, which demonstrates the trend in the behavior. Note, that formation of NCs was confirmed (Fig. 3) using high-resolution transmission electron microscopy (HRTEM). The PLQYs increase from 3.69% to 95.2% as substituting Cl with Br and I, opposite to the change in exciton average lifetime. The such phenomenon reflects that non-radiation losses reduce and lattice defects decrease, especially strongly for Br and I ion-containing PV materials.

The emission peaks can be further transferred into the color coordinates in Commission Internationale de L'Eclariage (CIE) 1931. The detailed parameters are summarized in Table S1 and Fig. S1.† It should be noted that CIE 1931 cites similar chromaticity coordinates for CsCu_2_X_3_ and Cs_3_Cu_2_X_5_ (X = Cl, Br, I) with solution-process-based PVs under 325 nm UV excitation at room temperature (RT).^15^

Moreover, to study the carrier dynamics of these six PV NC films on paper the TRPL measurement results (Fig. 2) were analyzed.

The average photogenerated carrier's lifetime profiles can be fitted using one or two exponential functions^36^
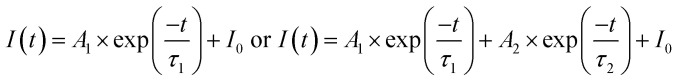


The results demonstrate that the average lifetime of these PVs synthesized using the solid-state method can be about 0.1 microsecond scale. For example, the average lifetime in CsCu_2_Cl_3_ is about 111.065 μs. However, by substituting Cl with Br and I, the average lifetime decreases to 0.631 μs. Similar behavior is obtained for Cs_3_Cu_2_X_5_ NC materials. It should be emphasized that the exciton lifetime of solid-state process-based NC materials is longer compared to the exciton lifetime registered in lead-free PVs synthesized using the simple solution process.^31^

To analyze the carrier lifetime decay of each element in these PVs, the X-ray photoelectron spectroscopy (XPS) of full spectra was further performed. The peak position of Cs 3d and I 3d had a greater shift than those of Cl 2p and Br 3d, as shown in Fig. S2–S7,† in respect to the same electron binding energies of Cu^+^. A similar trend is also observed for Cs_3_Cu_2_X_5_ single crystals, indicating the photoelectrical behavior of metal lead-free halide PVs is determined by halide ions. It should be noted that the atomic radius of Br and I atoms is larger compared to the atomic radius of Cl atom, therefore the atoms have more strong influence on carrier lifetime than Cl atom. In addition, solid-state phase lead-free metal halide PVs: CsCu_2_X_3_ and Cs_3_Cu_2_X_5_ NC materials exhibit higher stability in the air compared to those materials obtained using the solution phase.^7,29^

Furthermore, two Cu peaks of CsCu_2_X_3_ at 932.2 eV and 957.1 eV were resolved in the XPS spectra, which can be assigned to Cu 2p_2/3_ and Cu 2p_1/2_, respectively. Both Cu peaks form in [Cu_2_X_3_]^1−^ anion sites, the [CuX_3_]^1−^ is closer to the Cu^+^ state rather than the Cu^2+^ state,^37^ as shown in Fig. S2–S4.† However, Cu 2p_2/3_ and Cu 2p_1/2_ with a high binding energy of 931.4 eV and 958.2 eV related to the Cu 2p_2/3_ and Cu 2p_1/2_, and the Cu 2p signals in Cs_3_Cu_2_X_5_ NCs should be attributed to the valence of Cu^+^.^7^ Besides, the higher 933.2 and 953.7 eV, observed in Cs_3_Cu_2_X_5_, and the [Cu_2_X_5_]^3−^ are closer to the Cu^2+^ state rather than the Cu^+^ state.^37^ This results in existing two states for the Cu 2p spectra of Cs_3_Cu_2_X_5_ (Fig. S5–S7†).^14^

Meanwhile, each PV NC material exhibits a similar XPS spectrum with the core levels of Cs 3d and Cu 2p, but different core levels of Cl 2p, Br 3d and I 3d. For example, CsCu_2_Cl_3_ exhibited a characteristic peak at ∼200 eV (Cl 2p), but no Br 3d and I 3d related peaks are observed.^16^ A similar tendency is also observed for Cs_3_Cu_2_X_5_ single crystals,^21^ which is in good agreement with the results of energy dispersive X-ray spectroscopy (EDS).

The element composition was subsequently confirmed by EDS, as shown in Fig. S8–S13.† All elements (Cs, Cu, Cl, Br, I) were uniformly distributed in the selected area of NC films. The corresponding atomic ratios are also evaluated, confirming the stoichiometry of the formed perovskite NC films.

The High-Resolution TEM (HRTEM) images and Fast Fourier Transform (FFT) patterns of CsCu_2_X_3_ and Cs_3_Cu_2_X_5_ are shown in Fig. 3. Next, the evolution of the morphology CsCu_2_X_3_ and Cs_3_Cu_2_X_5_ NC materials is recorded as a function of different reaction times. Images of fabricated materials were obtained after reaction time at high temperatures: 400 °C and 550 °C (only for CsCu_2_Br_3_). With gradually increasing the reaction time from 2 h to 6 h, all PV NCs became larger in size. It is worth noting that some circle- and square-shaped nanosheets of the CsCu_2_Cl_3_ and Cs_3_Cu_2_Cl_5_ formed (Fig. S14a–d†). On the other hand, both CsCu_2_Br_3_ NCs and Cs_3_Cu_2_Br_5_ NC materials are gradually connecting with each other. These processes allow forming of nanowires or micro-crosses (Fig. S14e–h†). Notably, the morphologies of CsCu_2_I_3_ do not change with a reaction time of 4 h (Fig. S15i and j†), however, nanorods can be formed after reacting for 6 h (Fig. S15e†). For the Cs_3_Cu_2_I_5_, the morphologies are controllable at whole reaction stages, and the NCs are uniform (Fig. S14k and l†). The final morphology after 6 h solid-phase synthesis of PVs is studied using TEM, the results are shown in Fig. S15.† The proposed solid-state phase method (at 400 °C and at 550 °C – only for CsCu_2_Br_3_ material) for the fabrication of NC materials demonstrates higher stability compared to stabilities published in literature^29^ with greatly reduced surface recombination rate.

**Fig. 4:**
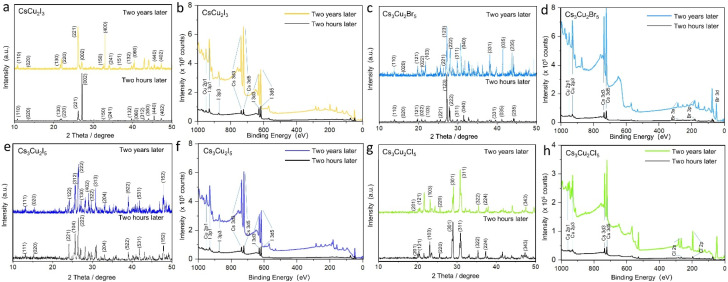
Powder XRD and XPS pattern obtained for different time periods: as prepared (black) and after two years (color curves): (a and b) orthorhombic CsCu_2_I_3_ and (c and d) Cs_3_Cu_2_I_5_; (e and f) Cs_3_Cu_2_Br_5_ and (g and h) Cs_3_Cu_2_Cl_5_ NC materials. The results demonstrate good crystallinity and stability of the PV materials.

To investigate the crystal phases of as-prepared perovskite NCs, powder X-ray diffraction (PXRD) was performed (Fig. 3). Each PV NC exhibited a unique PXRD pattern with distinctive characteristic peaks, confirming its crystal phase. The PXRD patterns of solid-state synthesis's Cs_3_Cu_2_X_5_ are in good agreement with the experiment data of Lian *et al.*^7^ The crystal structure of Cs_3_Cu_2_X_5_ species belongs to the space group *Pnma*, adopting an orthorhombic zero-dimensional structure. Besides, tetrahedral or triangular [Cu_2_X_5_]^3−^ anion sites are isolated by cesium (Cs^+^) cations. For the case of CsCu_2_Br_3_ and Cs_3_Cu_2_Br_5_, the PXRD pattern shows orthorhombic phase with distinguished peaks: (311) and (221) in Fig. 3b, (222) and (123) in Fig. 3e, respectively. The PXRD spectrum of CsCu_2_X_3_ nanosheets does not suffer severe peak broadening, therefore this allows easier distinguishing their phase from Cs_3_Cu_2_X_5_ species. For instance, the one-dimensional electronic structure CsCu_2_X_3_ group belongs to the orthorhombic space group *Cmcm*, and the [Cu_2_X_3_]^3−^ anion sites are also separated by rows of Cs^+^ cations.^15^ In addition, these PVs exhibit a high thermal resistance after treatment at a high temperature (400 °C or 550 °C), as well as good environmental stability after storing 60 days (Table S1†). The PXRD and XPS spectra recorded for NC PVs after two years (shown in Fig. 4) demonstrate that most of the materials have good crystallinity and reproducibility still. It should be emphasized, all PV NC powders have been stored in exposed-to-air transparent glass reagent bottles. These PVs materials have been obtained on a conductive tape of 0.3 inch discretely with PV thin-film thickness of about 2 μm. The powders stored for two years show reproducible XRD and XPS results. The fact confirms the high quality of obtained lead-free PVs.

**Fig. 5:**
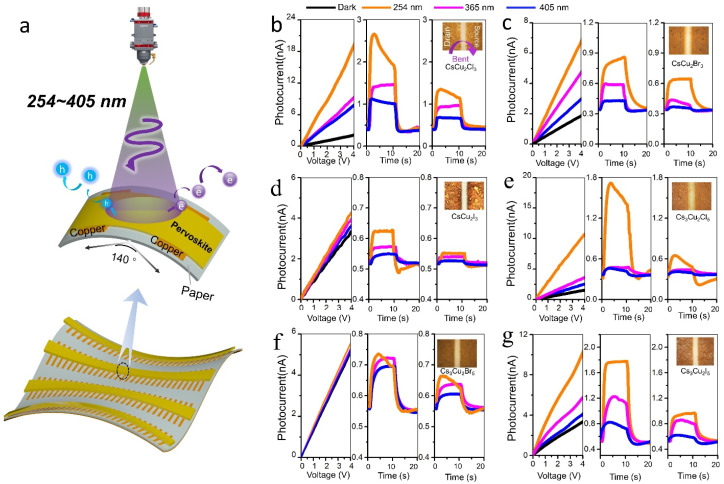
(a) Schematic diagram showing the device structure consisting of paper/perovskite/copper. Photocurrent output characteristics and on–off photocurrent ratio at a bias voltage of 5 mV with 10 μW cm^−2^ power intensities of the (b) CsCu_2_Cl_3_ (c) Cs_3_Cu_2_Cl_5_ (d) CsCu_2_Br_3_ (e) Cs_3_Cu_2_Br_5_ (f) CsCu_2_I_3_ (g) Cs_3_Cu_2_I_5_ film devices with various wavelengths irradiation 254, 365 and 405 nm laser intensities with (w) bent or without (w/o) bent, respectively. Insets: digital photographs of the paper-based photodetector with a channel width of 500 μm and length of 100 μm between the drain and source copper electrodes.

To deeply study all-inorganic lead-free PVs, flexible photodetectors based on these materials were fabricated using the paper substrate, as schematically illustrated in Fig. 5a. The copper electrodes are deposited by electron beam evaporation with a distance of 100 μm. The photodetectors exhibit obvious photoresponse at a fixed 40° angle after hundreds of bending under different wavelengths (254, 365, and 405 nm) irradiation, as shown in Fig. 5. After returning to a flat state, the optical and electrical performance can be recovered as before. The bandgap (*E*_g_) can be subsequently evaluated by ultraviolet-visible (UV-Vis) absorption spectroscopy (Fig. S16†) and using an empirical formula *E*_g_ = *hc*/*λ*, where is the plank constant, *c* denotes the velocity of light, and *λ* is the cut-off wavelength. The *E*_g_ can be determined as 5.02 eV, 4.96 eV and 4.49 eV for CsCu_2_Cl_3_, CsCu_2_Br_3_ and CsCu_2_I_3_, 2.12 eV, 3.23 eV and 3.30 eV for Cs_3_Cu_2_Cl_5_, Cs_3_Cu_2_Br_5_ and Cs_3_Cu_2_I_5_ crystals, respectively. Since the valence band and conduction band are mainly formed due to the contribution of halides (Cl, Br, I) and Cu 2p orbits, respectively, and these halide and copper atoms are distributed around the hexagonal structure, therefore Cs ions have little influence on the electronic structure of band edge in the middle of the hexagonal structure.

Noteworthily, devices fabricated based on large *E*_g_ perovskites are not sensitive to 405 nm and even shorter wavelength irradiation. This can be explained as follows. The excited electrons at 254, 365 and 405 nm wavelengths irradiation could not spill over into the photon-emitting state for CsCu_2_I_3_*E*_g_ of 4.49 eV and Cs_3_Cu_2_Br_5_*E*_g_ of 3.23 eV.

To further explore the photoelectrical properties of these devices, the output characteristics and on/off current transient photoresponse of the photodetectors were recorded (Fig. 5b–g). All devices exhibit reproducible on/off cycles, indicating good electrical stability. In addition, the output photoresponse current characteristics were found to be strongly dependent upon the wavelength of the incident illumination. The fact indicates a good wavelength selectivity. The on/off ratio of the CsCu_2_X_3_ and Cs_3_Cu_2_X_5_ photodetectors are subsequently measured at 10 μW cm^−2^ and bias voltage (*V*_DS_) 5 mV, and summarized in Table 1, as a function of exciting wavelength.

**Table 1:** Characteristics of CsCuX-based UV detectors, measured at 10 μW cm^−2^ photon power density and *V*_DS_ = 5 mV before and after the bending experiment

Materials	Wavelength (nm)	*R* (A W^−1^) w/o bent	*R* (A W^−1^) w bent	D* (jones) w/o bent	D* (jones) w bent
CsCu_2_Cl_3_	254	1.10 × 10^−3^	3.76 × 10^−4^	7.46 × 10^9^	2.71 × 10^9^
	365	4.36 × 10^−4^	2.11 × 10^−4^	2.96 × 10^9^	1.52 × 10^9^
	405	3.20 × 10^−4^	1.40 × 10^−4^	2.17 × 10^9^	1.01 × 10^9^
Cs_3_Cu_2_Cl_5_	254	6.84 × 10^−4^	2.66 × 10^−4^	4.51 × 10^9^	1.75 × 10^9^
	365	1.92 × 10^−4^	1.77 × 10^−4^	1.26 × 10^9^	1.17 × 10^9^
	405	1.84 × 10^−4^	2.66 × 10^−4^	1.21 × 10^9^	1.75 × 10^9^
CsCu_2_Br_3_	254	3.44 × 10^−4^	2.56 × 10^−4^	2.40 × 10^9^	1.79 × 10^9^
	365	2.35 × 10^−4^	1.76 × 10^−4^	1.65 × 10^9^	1.23 × 10^9^
	405	1.72 × 10^−4^	1.48 × 10^−4^	1.20 × 10^9^	1.03 × 10^9^
Cs_3_Cu_2_Br_5_	254	2.96 × 10^−4^	2.64 × 10^−4^	1.59 × 10^9^	1.41 × 10^9^
	365	2.88 × 10^−4^	2.56 × 10^−4^	1.54 × 10^9^	1.37 × 10^9^
	405	2.76 × 10^−4^	2.41 × 10^−4^	1.48 × 10^9^	1.29 × 10^9^
CsCu_2_I_3_	254	2.52 × 10^−4^	2.23 × 10^−4^	1.39 × 10^9^	1.23 × 10^9^
	365	2.28 × 10^−4^	2.16 × 10^−4^	1.26 × 10^9^	1.20 × 10^9^
	405	2.20 × 10^−4^	2.12 × 10^−4^	1.22 × 10^9^	1.17 × 10^9^
Cs_3_Cu_2_I_5_	254	3.28 × 10^−4^	2.48 × 10^−4^	3.92 × 10^9^	2.14 × 10^9^
	365	4.84 × 10^−4^	3.54 × 10^−4^	2.68 × 10^9^	1.96 × 10^9^
	405	7.08 × 10^−4^	3.87 × 10^−4^	1.82 × 10^9^	1.37 × 10^9^

The *R* and D* decreased after the bending with a bending angle of 40° and recorded in Table 1 (the bent direction is parallel to the arrow in Fig. 5b), respectively. The bending of the absorbing surface changes the incident angle and area of the light, which reduced the projected area of light on the PV photodetector. Although the on-off ratio and the response speed of the perovskite devices are far from the desired state-of-art photoelectric application, all these results indicated that the photodetectors using the solid-state method may exhibit good near ultraviolet photoresponse, as well as stable and reproducible photo-switching characteristic as it is shown in this work. It should be noted that photovoltaic properties, allowing to capture of solar energy will enable the future fabrication of wearable biosensors, which do not require a battery. The devices fabricated on the flexible substrate will enable the exploration of new pathways for delivering approaches to create highly functional skin-integrated biosensors employing photovoltaic techniques that are capable of working on renewable energy sources, reducing the cost in a green energy and technology strategy. These innovations may be quickly implemented by the industry for commercial production to bring high-speed, safe and effective biomedical devices to market more quickly and at a cost-efficient basis, to play a major role in solving global challenges in an environmentally-friendly and sustainable way.

## Experimental

### Materials

The following commercial materials were utilized without further purification unless otherwise indicated. CsBr (99.998%, Xi'an Polymer Light Technology Corp, China), CsBr (99.993%, Xi'an Polymer Light Technology Corp, China), CsI (99.997%, Xi'an Polymer Light Technology Corp, China), CuI (97%, Aladdin), CuBr (98%, Aladdin), CuCl (≥96%) and *N*,*N*-dimethylformamide (DMF, ≥99%, Sigma-Aldrich).

### Synthesis of NC materials with the solid-state method

All perovskites were prepared by reacting a 1 : 2, 3 : 2 stoichiometric ratio of CsX : CuX (X = Cl, Br, I) reactants. To ensure the homogeneity of all studied perovskite precursors, all reactants were ground in zirconia ball mill jars and then sealed in evacuated quartz ampules. Reaction mixtures were then, after the optimization, annealed at 400 °C (or 550 °C only CsCu_2_Br_3_ NC material) for 6 h (raise step is 5 °C per min from RT for 4.5 h) and natural cooling to room temperature for 5 h. As-prepared 200 mg perovskite powders (200 mg) were subsequently dissolved in a DMF (50 mL) under 200 °C heating. To fabricate a high-quality perovskite thin film, the perovskite solution was spin-coated on a paper-based substrate with a rotation speed of 1200 rpm for 35 s, followed by annealing at 100 °C for 15 min. The DMF will evaporate from PVs thin films. The Cu electrode with a thickness of 400 nm was deposited using EBV technology and then the devices with sub-micrometer characteristic size were formed.

### Material characterization

The morphology of samples is analyzed using SEM (FEI NanoSEM 50). Chemical identification of perovskite NCs was conducted by XRD (MAXima XRD-7000). The micro-level structure is characterized by TEM (FEI Tecnai G2F20 S-Twin). For the elemental analysis of the as-synthesized perovskite NCs, XPS data are obtained on a Kratos AXIS-ULTRA DLD photoelectron spectrometer. UV/Vis absorption spectra are obtained using a PerkinElmer UV WinLab, spectrophotometer. PLQYs measurements were performed using HAMAMATSU Quantaurus-QY Plus UV-NIR. The fluorescence lifetime image and excitation spectra were employed for Edinburgh FLS1000 spectrofluorometer at RT. Reflection spectra were obtained by a Shimadzu UV2600 with the external integrating sphere at RT.

### Device characterization

The direct current characteristics *I*–*V* curves of the perovskite photodetector were carried out using an Agilent parameter analyzer B1500a. The laser diode is controlled to produce a square wave light pulse by a programmable power source Agilent E3640A.

## Conclusions

In summary, facile scale-up synthesis of Cs copper halide NC materials using the solid-state method developed in this work is reported. Such perovskite NCs not only exhibited a good thermal stability after storing in an air environment for two years but also high PLQYs reaching up to 90%. Moreover, a long carrier lifetime of ∼116 μs was obtained, showing a good crystal quality of synthesized NC materials. The flexible photodetectors based on these materials undoubtedly exhibited a good photoresponse to UV light, including a responsivity of above 1.0 × 10^−4^ A W^−1^, a detectivity of 1.0 × 10^9^ jones under such low photon power density (10 μW cm^−2^) and *V*_DS_ = 5 mV. Finally, good air stability and photoswitching characteristics achieved for the photodetectors on flexible substrates show great potential for future flexible electronics, including bioelectronics and solar energy-powered biosensors on the flexible substrate.

## Author contributions

Zhi Jiang conceived the idea of scale-up perovskite synthesis and assembled photodetector on flexible paper. Hezhuang Liu and Jihua Zou verified the analytical methods. Yixuan Huang contributed to the interpretation of the results. Zhaoquan Xu and Denys Pustovyi carried out the experiment and contributed to the presentation of the original research data; Svetlana Vitusevich contributed to the writing and editing of the paper. All authors have read, contributed to the editing and agreed on the present version of the manuscript.

## Conflicts of interest

There are no conflicts to declare.

## Supplementary Material

RA-013-D2RA07100B-s001

RA-013-D2RA07100B-s002
