# Supplementary material for: Scale-up synthesis of high-quality solid-state-processed CsCuX (X = Cl, Br, I) perovskite nanocrystal materials toward near-ultraviolet flexible electronic properties
Source: RSC Adv. 2023 Feb 20;13(9):5993–6001. doi: 10.1039/d2ra07100b (PMC9939939; doi:10.1039/d2ra07100b)
Supplement: RA-013-D2RA07100B-s001 [file RA-013-D2RA07100B-s001.pdf]

Electronic Supplementary Material (ESI) for RSC Advances.
This journal is © The Royal Society of Chemistry 2023

Solid-State Synthesis of Lead-Free Perovskite Materials Toward Near-Ultraviolet Flexible Electronics

Zhi Jiang, [a,b] Hezhuang Liu, [b] Jihua Zou, [b] Yixuan Huang, [b] Zhaoquan Xu, [b] Denys Pustovyi[a] and Svetlana Vitusevich[a]

[a] Institute of Biological Information Processing (IBI-3), Forschungszentrum Jülich, Juelich 52425, Germany

[b] Institute of Fundamental and Frontier Sciences, University of Electronic Science and Technology of China, Chengdu 610054, China

‡‡Authors to whom correspondence should be addressed: Jiang, Zhi z.jiang@fz-juelich.de, Svetlana Vitusevich s.vitusevich@fz-juelich.de

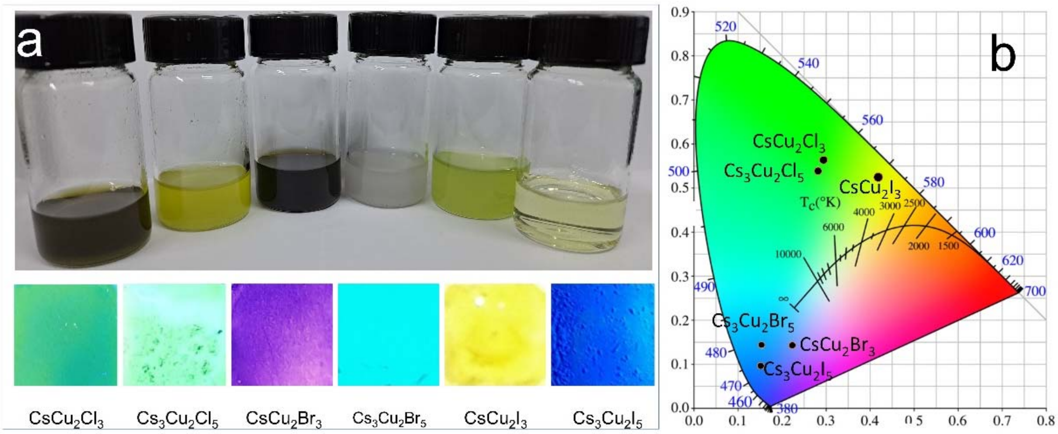

Figure S1. (a) The digital images of $CsCu_2Cl_3$, $Cs_3Cu_2Cl_5$, $CsCu_2Br_3$, $Cs_3Cu_2Br_5$, $CsCu_2I_3$ and $Cs_3Cu_2I_5$ dissolved into DMF. Insets (below the images) demonstrate the corresponding PL emission on paper substrate. (b) CIE 1931 chromaticity plot of the six lead-free halides perovskite.

Table S1. Photophysical properties of $Cs_3Cu_2X_5$ perovskite, exposed to Air for 60 days

Materials	λ_{ex} (nm)	λ_{em} (nm)	CIE	lifetime (μs)	PLQY(%)
$CsCu_2Cl_3$	308	530	(0.2938, 0.5643)	111.065	3.69
$Cs_3Cu_2Cl_5$	312	495	(0.2813, 0.5424)	109.815	4.26
$CsCu_2Br_3$	315	435	(0.1814, 0.1542)	5.433	9.56
$Cs_3Cu_2Br_5$	310	460	(0.155, 0.1415)	20.668	20.22
$CsCu_2I_3$	334	560	(0.4156, 0.5267)	0.631	22.81
$Cs_3Cu_2I_5$	320	445	(0.1513 0.0978)	1.002	95.20

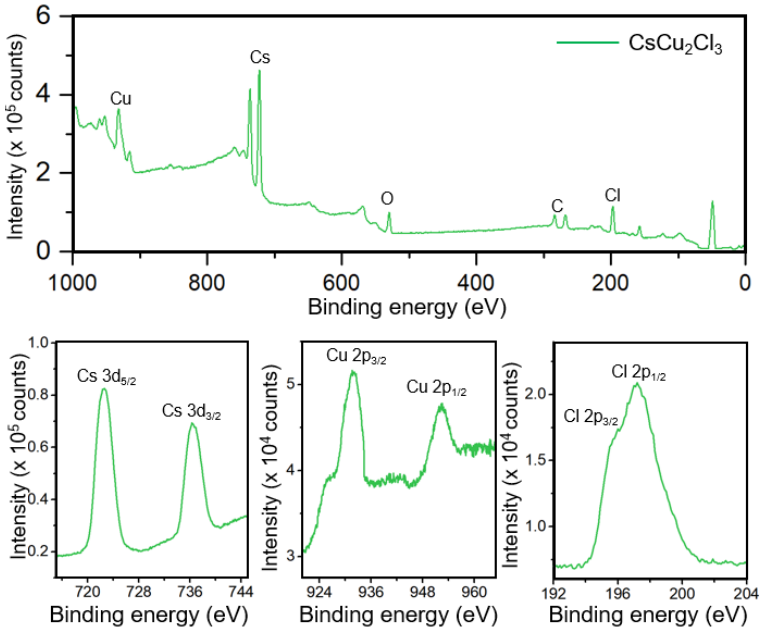

Figure S2. XPS spectra of $CsCu_2Cl_3$ powders.

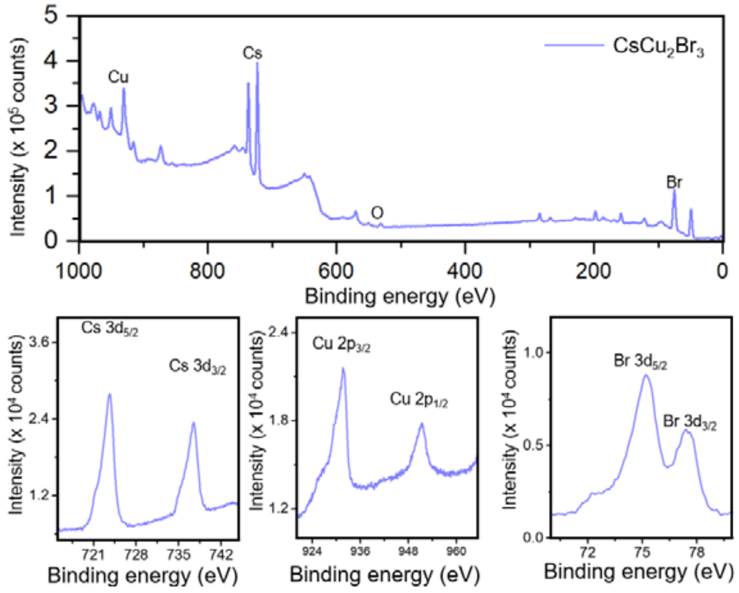

Figure S3. XPS spectra of $CsCu_2Br_3$ powders.

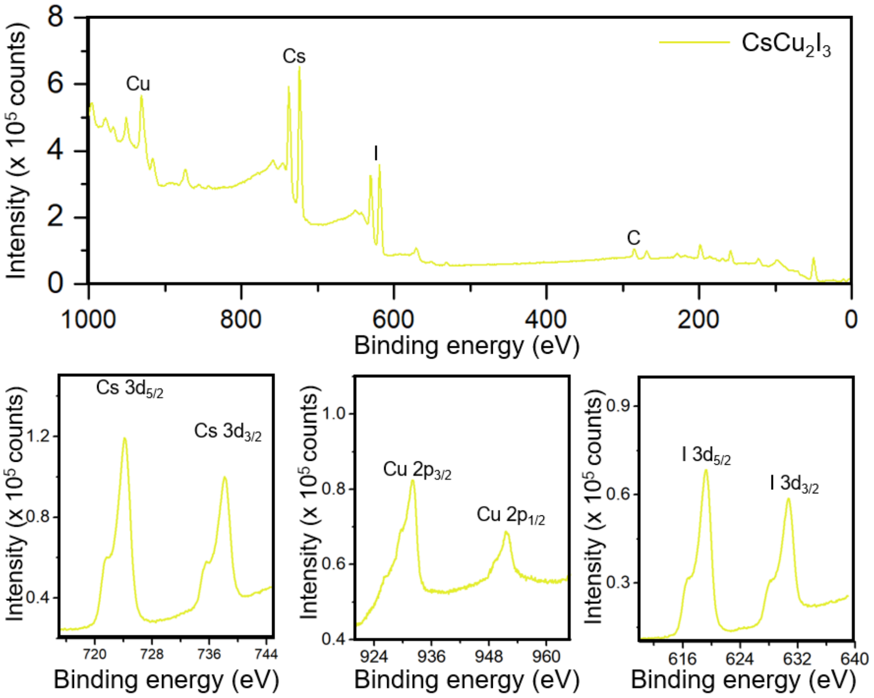

Figure S4. XPS spectra of $CsCu_2I_3$ powders.

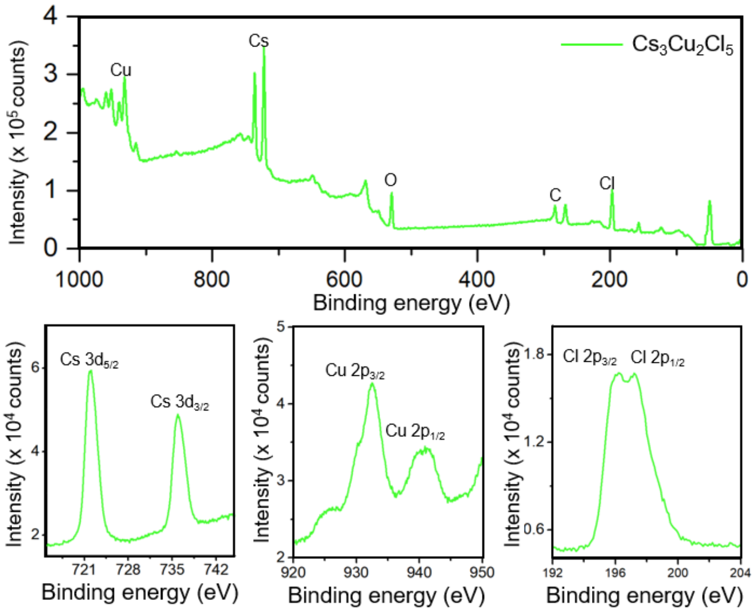

Figure S5. XPS spectra of $Cs_3Cu_2Cl_5$ powders.

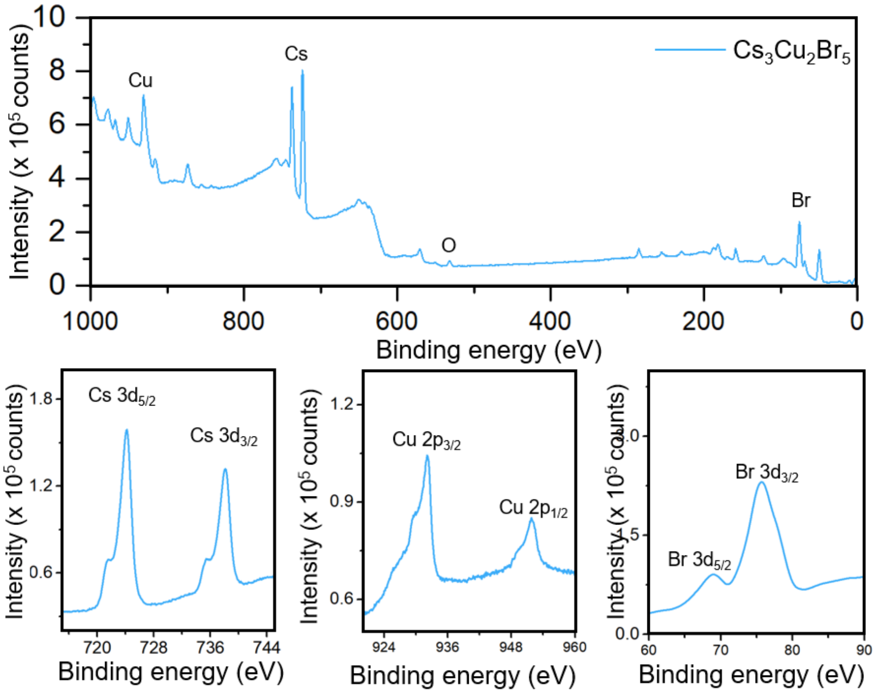

Figure S6. XPS spectra of $Cs_3Cu_2Br_5$ powders.

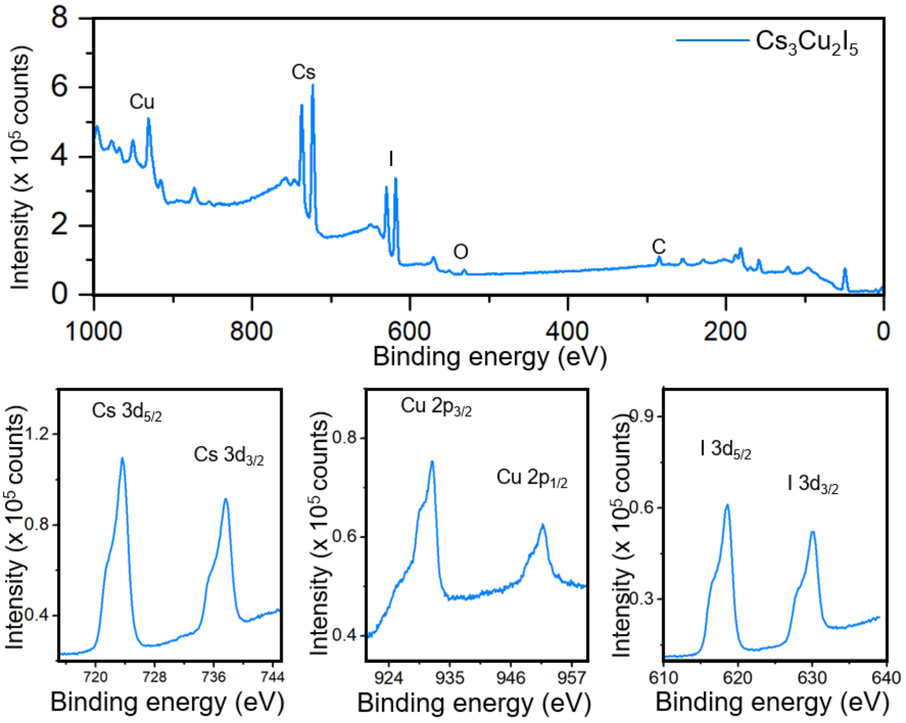

Figure S7. XPS spectra of $Cs_3Cu_2I_5$ powders.

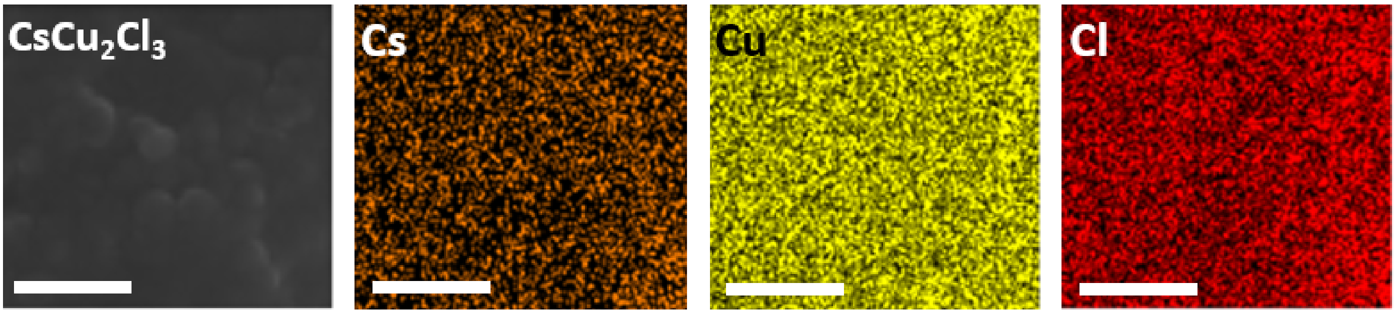

Element	Line Type	Apparent Concentration	k Ratio	Wt%	Wt% Sigma	Atomic %
Cl	L series	15.97	0.12875	31.56	0.34	48.42
Cu	L series	14.73	0.14967	33.86	0.52	35.85
Cs	L series	12.18	0.12177	34.58	0.68	15.73
Total:				100.00		100.00

Figure S8. SEM images (top) of **$CsCu_2Cl_3$** thin film on paper, the scale bar is 500 nm. The colors reflect different material content in pvs. The element EDS results summarized also in the Table below obtained for the corresponding perovskite films on paper.

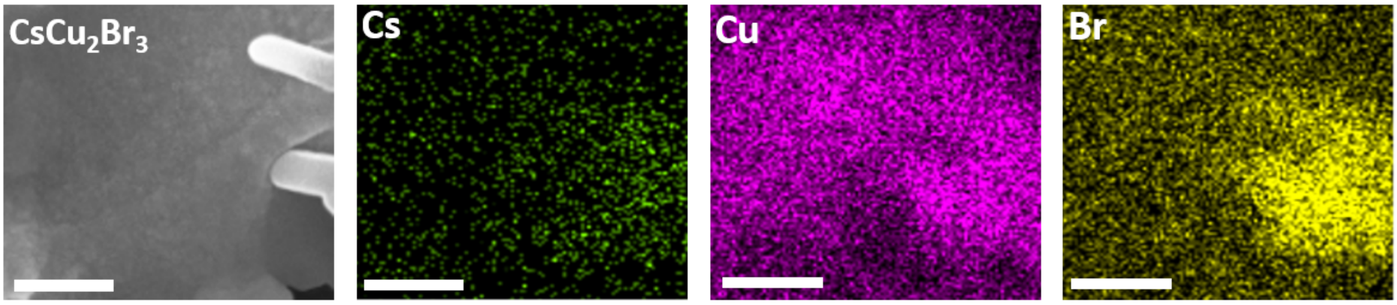

Element	Line Type	Apparent Concentration	k Ratio	Wt%	Wt% Sigma	Atomic %
Br	L series	38.51	0.38514	43.37	0.58	53.91
Cu	L series	22.42	0.20073	13.52	0.49	30.05
Cs	L series	26.58	0.05010	43.11	0.75	16.04
Total:				100.00		100.00

Figure S9. SEM image (top on the left) of **$CsCu_2Br_3$** thin film on paper, the scale bar is 500 nm. The element EDS results of the corresponding perovskite films on paper.

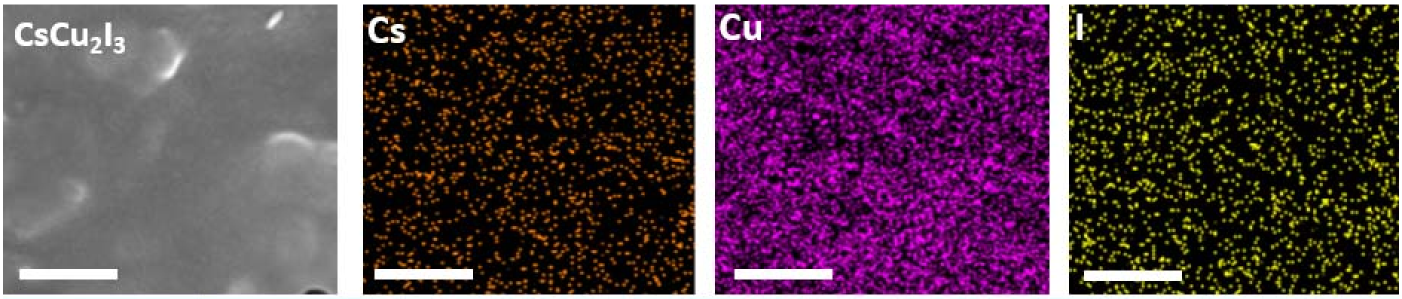

Element	Line Type	Apparent Concentration	k Ratio	Wt%	Wt% Sigma	Atomic %
I	L series	12.63	0.08075	59.58	1.42	35.66
Cu	L series	4.07	0.16631	19.91	1.82	50.78
Cs	L series	4.97	0.02969	20.51	1.94	13.56
Total:				100.00		100.00

Figure S10. SEM image (top on the left) of **$CsCu_2I_3$** thin film on paper, the scale bar is 500 nm. The element EDS results summarized also in the Table below obtained for the corresponding perovskite films on paper.

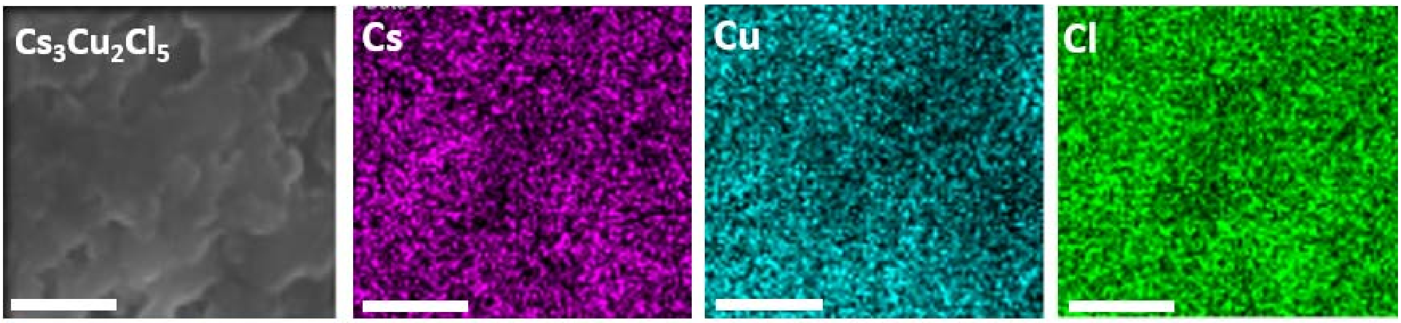

Element	Line Type	Apparent Concentration	k Ratio	Wt%	Wt% Sigma	Atomic %
Cl	K series	40.81	0.35665	23.87	0.26	46.91
Cu	L series	19.27	0.19271	23.00	0.31	25.23
Cs	L series	63.39	0.63392	53.13	0.43	27.86
Total:				100.00		100.00

Figure S11. SEM image (top on the left) of **$Cs_3Cu_2Cl_5$** thin film on paper, the scale bar is 500 nm. The element EDS results summarized also in the Table below obtained for the corresponding perovskite films on paper.

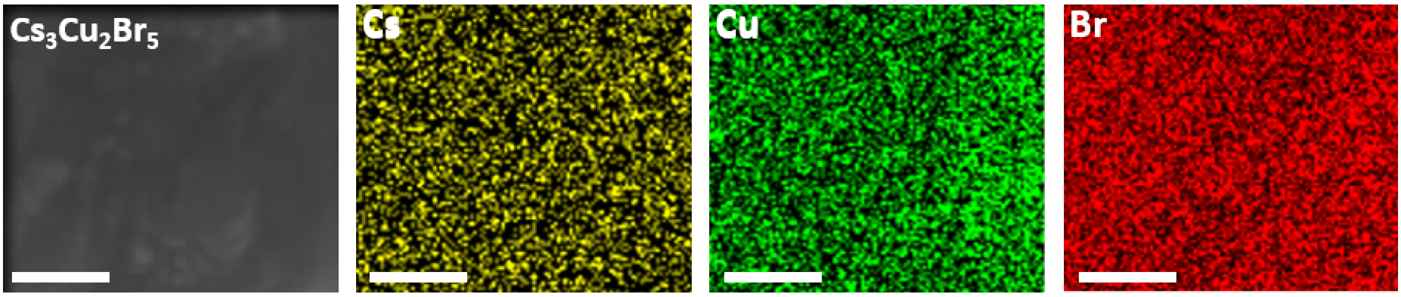

Element	Line Type	Apparent Concentration	k Ratio	Wt%	Wt% Sigma	Atomic %
Br	L series	58.98	0.52817	42.67	0.51	49.38
Cu	L series	15.29	0.15294	14.12	0.32	20.56
Cs	L series	63.50	0.63501	43.20	0.59	30.06
Total:				100.00		100.00

Figure S12. SEM image (top on the left) of $\mathbf{Cs_3Cu_2Br_5}$ thin film on paper, the scale bar is 500 nm. The element EDS results summarized also in the Table below obtained for the corresponding perovskite films on paper.

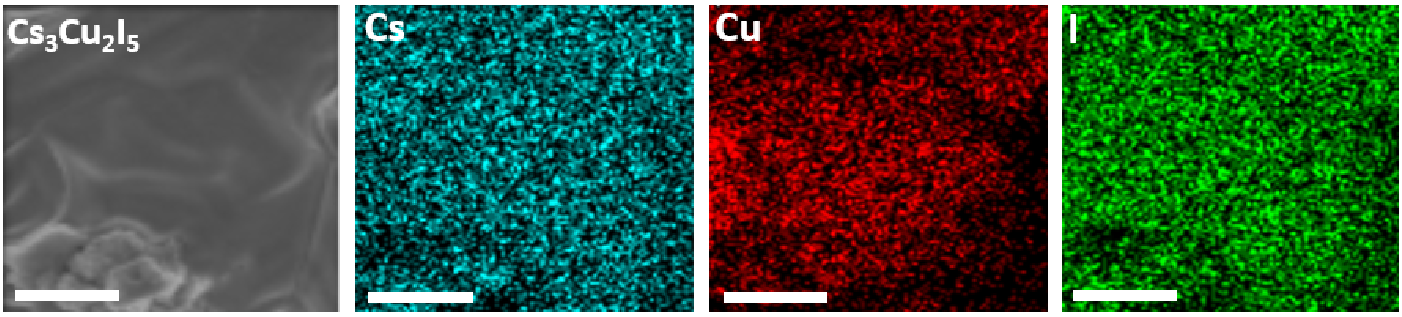

Element	Line Type	Apparent Concentration	k Ratio	Wt%	Wt% Sigma	Atomic %
I	L series	94.23	0.94229	54.20	0.60	48.97
Cu	L series	10.94	0.10938	12.24	0.31	22.09
Cs	L series	57.83	0.57827	33.55	0.63	28.94
Total:				100.00		100.00

Figure S13. SEM image (top on the left) of $\mathbf{Cs_3Cu_2I_5}$ thin film on paper, the scale bar is 500 nm. The element EDS results summarized also in the Table below obtained for the corresponding perovskite films on paper.

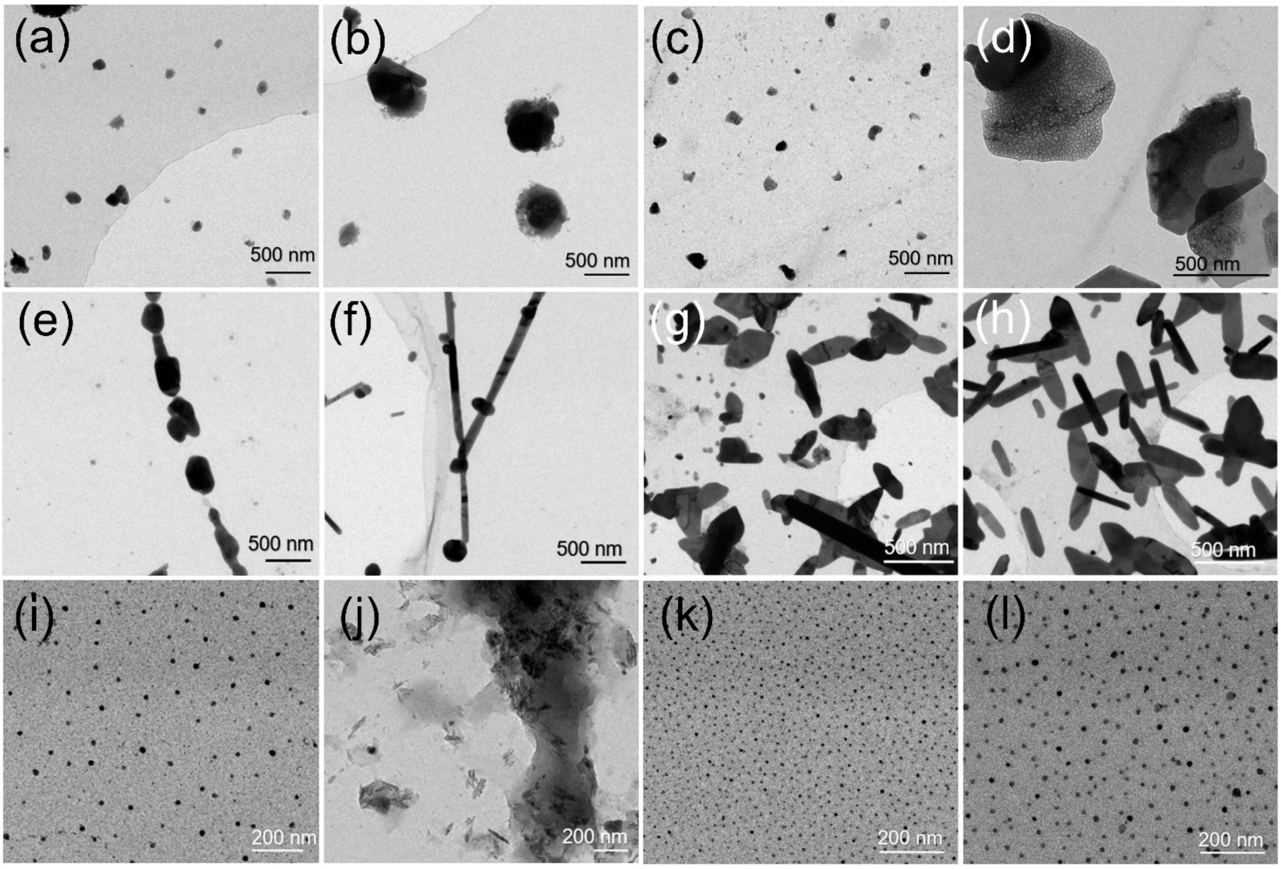

Figure S14. Morphology and shape evolution of the as-prepared $CsCu_2X_3$ and $Cs_3Cu_2X_5$ NCs synthesized with different reaction time. 2h and 4h; (a) and (b) for **$CsCu_2Cl_3$**, (c) and (d) for **$Cs_3Cu_2Cl_5$**, (e) and (f) for **$CsCu_2Br_3$**, (g) and (h) for **$Cs_3Cu_2Br_5$**, (i) and (j) for **$CsCu_2I_3$**, (k) and (l) for **$Cs_3Cu_2I_5$**, respectively

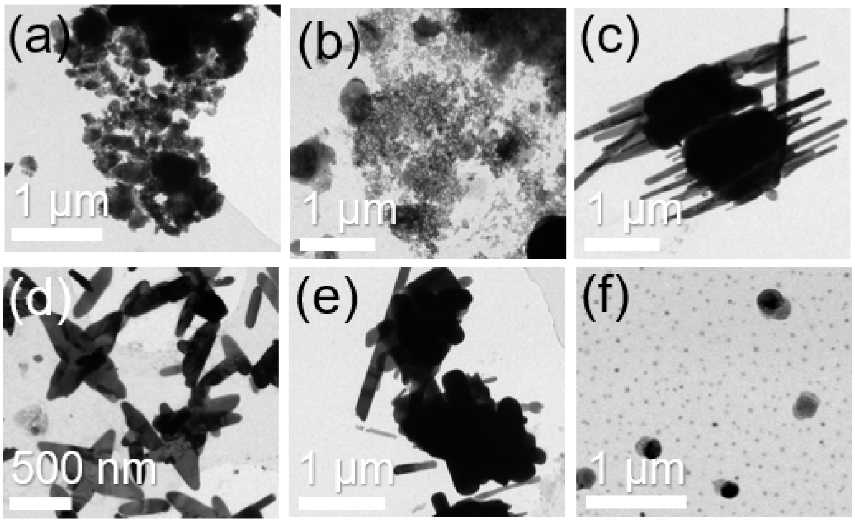

Figure S15. TEM images of (a) $CsCu_2Cl_3$ (b) $Cs_3Cu_2Cl_5$ (c)$CsCu_2Br_3$ (d) $Cs_3Cu_2Br_5$ (e) $CsCu_2I_3$ (f) $Cs_3Cu_2I_5$ obtained for the case of 6h reaction time.

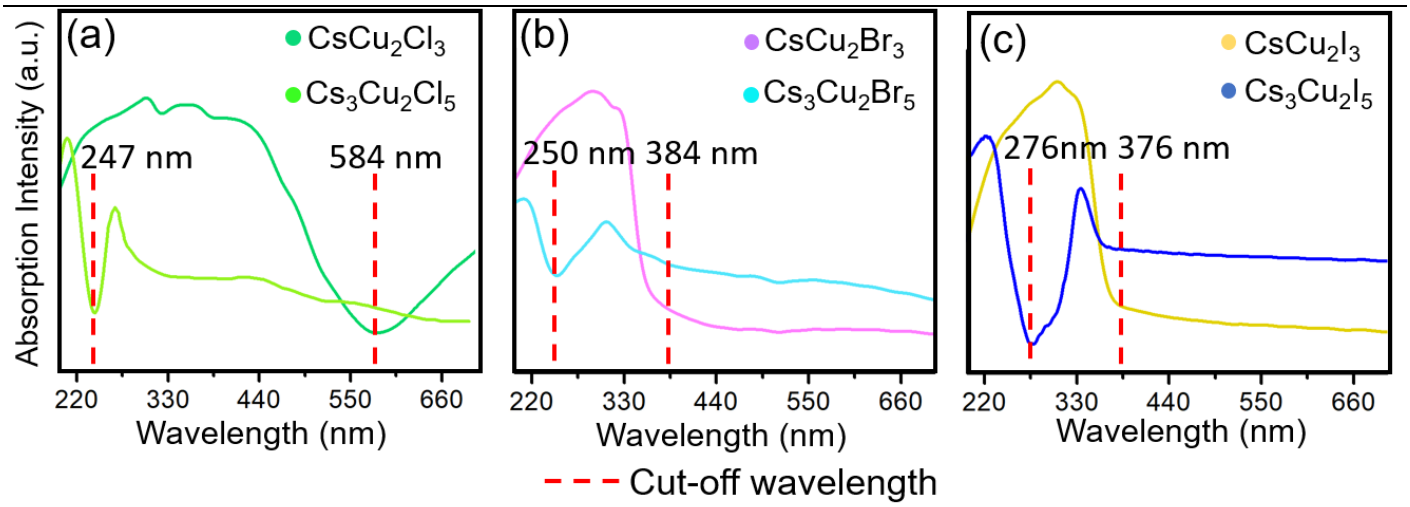

Figure S16. UV/Vis absorption spectra of (a) $CsCu_2Cl_3$ and $Cs_3Cu_2Cl_5$, (b) $CsCu_2Br_3$ and $Cs_3Cu_2Br_5$, (c) $CsCu_2I_3$ and $Cs_3Cu_2I_5$ crystals.
